# Small extracellular vesicles, their cargo, and oxidative stress: emerging biomarkers for glaucoma diagnosis and treatment

**DOI:** 10.20517/evcna.2025.22

**Published:** 2025-05-28

**Authors:** Francisco J. Romero, Manuel Diaz-Llopis

**Affiliations:** ^1^Department of Emergency, Requena General Hospital, Requena 46340, Spain.; ^2^MediPark Clinic, Olten 4600, Switzerland.; ^3^Department of Ophthalmology, School of Medicine and Dentistry, University of Valencia, Valencia 46010, Spain.

**Keywords:** Extracellular vesicles, glaucoma, oxidative stress, proteomics

## Abstract

The contribution commented on is a relevant bridging work between extracellular vesicles, oxidative stress, and proteomics (especially regarding biomarkers) research. The findings reported are promising but further point out one of the future directions for investigations in glaucoma and certainly other such complex diseases.

The study by Rejas-González *et al*., published in *Redox Biology*^[1]^, investigates the intricate relationship between oxidative stress, ocular diseases, and small extracellular vesicles (sEVs) to identify novel biomarkers for glaucoma. This commentary summarizes the research objectives, methodology, and implications while highlighting potential future directions and applications of the findings.

Oxidative stress is a well-established factor in glaucoma pathogenesis^[2-4]^. It disrupts cellular equilibrium, damages biomolecules, and impairs mitochondrial function^[5]^. Given the high sensitivity of ocular structures, such as the trabecular meshwork and retinal ganglion cells, to oxidative damage^[6]^, understanding this process is crucial. The study reinforces oxidative stress as a key contributor to glaucoma and suggests novel diagnostic avenues.

Although numerous studies have linked oxidative stress to glaucoma, its molecular mechanisms and clinical significance remain insufficiently understood. By incorporating sEV analysis, this research goes beyond traditional biomarkers to explore vesicle-associated proteins as indicators of oxidative stress-related changes^[7]^. This innovative approach aligns with emerging trends in biomarker discovery.

sEVs have emerged as valuable diagnostic and therapeutic tools due to their capacity to transport biomolecules, including proteins, lipids, and RNAs, from their parent cells. They serve as key mediators of intercellular communication and can reflect cellular health or dysfunction (for a recent review in retinal diseases cf. Romero *et al*.^[8]^). In glaucoma, sEVs provide a non-invasive means to study cellular stress and damage in ocular tissues.

By isolating and analyzing sEVs from patient samples, the researchers identified distinct proteomic signatures associated with glaucoma. These profiles not only enhance our comprehension of disease-specific molecular pathways but also offer a non-invasive method for tracking disease progression and treatment response.

The discovery of oxidative stress-related proteins in sEVs is particularly significant. Proteins such as superoxide dismutase, peroxiredoxins, and heat shock proteins were identified as key players in glaucoma-related oxidative stress. These findings build on previous knowledge of oxidative stress in glaucoma while offering new insights into how these proteins are packaged and transported via sEVs.

Furthermore, the proteomic data serve as a valuable resource for further investigation. Detailed protein profiles may help identify new therapeutic targets or enable the development of combination biomarkers that enhance diagnostic accuracy. However, proteomic research presents challenges, including the need for validation in larger and more diverse patient populations.

A key contribution of this research is its potential clinical impact. Current glaucoma diagnostics primarily rely on functional and structural assessments, such as intraocular pressure measurements and optic nerve imaging. These techniques often detect glaucoma at advanced stages. The identification of sEV-based biomarkers presents an opportunity for earlier and more accurate diagnosis.

Additionally, the findings support a move toward personalized medicine. Individual proteomic profiles could allow clinicians to categorize patients based on their risk of disease progression or response to treatment. For example, individuals with high levels of oxidative stress-related proteins in their sEVs might benefit from targeted antioxidant therapies. Similarly, sEV biomarkers could help monitor treatment effectiveness, enabling more responsive and individualized care.

Another key consideration is the translational feasibility of these findings. Although the proteomic profiles identified in this study show promise, their clinical application depends on the development of reliable, cost-effective methods for sEV isolation and analysis. Standardizing protocols and validating biomarkers in independent cohorts are crucial next steps.

This study is aligned with broader research trends in ophthalmology and biomarker discovery. The use of EVs as biomarkers is gaining traction across fields such as oncology, neurology, and cardiology. Applying this approach to glaucoma research underscores the versatility of sEVs in diagnostics.

Additionally, the study integrates oxidative stress research with proteomics, bridging multiple disciplines and promoting interdisciplinary collaboration. Such integrative approaches are essential for tackling complex diseases like glaucoma.

Despite its strengths, the study has some limitations. The sample size and demographic diversity were not extensively detailed, which may limit the generalizability of the findings. Expanding the study cohort could enhance the robustness of the conclusions. Additionally, while the study demonstrates a strong association between sEVs and glaucoma, the functional consequences of the identified proteins require further investigation. Future studies should aim to determine their precise roles in disease onset and progression. In studies of this nature, it is advisable to use additional cell lines to validate the findings. Certainly, the R28 cell line is of retinal lineage^[9]^, but not specifically derived from ganglion cells. Thus, other cell lines such as RGC-5^[10]^ might be more suitable for confirming the results reported by the authors.

In conclusion, The article in *Redox Biology* presents compelling evidence for the potential of sEV-based biomarkers in the early detection and personalized treatment of glaucoma. While challenges remain, these findings mark an important advancement in glaucoma research and clinical practice. Future research should focus on validating these biomarkers across diverse populations, elucidating their functional roles, and developing practical methods for their clinical application. The integration of sEV biomarkers into glaucoma care has the potential to usher in a new era of precision medicine, ultimately improving patient outcomes worldwide.

## DECLARATIONS

### Copyright

© The Author(s) 2025.
